# Characterization of Feruloyl Esterase from *Bacillus pumilus* SK52.001 and Its Application in Ferulic Acid Production from De-Starched Wheat Bran

**DOI:** 10.3390/foods10061229

**Published:** 2021-05-28

**Authors:** Xiaoli Duan, Yiwei Dai, Tao Zhang

**Affiliations:** 1 State Key Laboratory of Food Science and Technology, Jiangnan University, Wuxi 214122, China; 6180112008@stu.jiangnan.edu.cn (X.D.); 7160112004@vip.jiangnan.edu.cn (Y.D.); 2 International Joint Laboratory on Food Safety, Jiangnan University, Wuxi 214122, China

**Keywords:** feruloyl esterase, *Bacillus pumilus*, characterization, synergistic effect

## Abstract

Feruloyl esterase (FAE; EC 3.1.1.73) catalyzes the hydrolysis of the 4-hydroxy-3-methoxycinnamoyl group in an esterified sugar to assist in waste biomass degradation or to release ferulic acid (FA). An FAE-producing strain was isolated from humus soil samples and identified as *Bacillus pumilus* SK52.001. The BpFAE gene from *B. pumilus* SK52.001 was speculated and heterogeneously expressed in *Bacillus subtilis* WB800 for the first time. The enzyme exists as a monomer with 303 amino acids and a molecular mass of 33.6 kDa. Its specific activity was 377.9 ± 10.3 U/(mg protein), using methyl ferulate as a substrate. It displays an optimal alkaline pH of 9.0, an optimal temperature of 50 °C, and half-lives of 1434, 327, 235, and 68 min at 50, 55, 60, and 65 °C, respectively. Moreover, the purified BpFAE released 4.98% FA of the alkali-acidic extractable FA from de-starched wheat bran (DSWB). When the DSWB was enzymatically degraded by the synergistic effect of the BpFAE and commercial xylanase, the FA amount reached 49.47%. It suggested that the alkaline BpFAE from *B. pumilus* SK52.001, which was heterologously expressed in *B. subtilis* WB800, possesses great potential for biomass degradation and achieving high-added value FA production from food by-products.

## 1. Introduction

Feruloyl esterase (FAE; EC 3.1.1.73) is a member of the alpha/beta hydrolase family that cleaves ester or ether linkages between ferulic acid (FA) and sugars in order to facilitate plant cell wall degradation and release FA [1,2]. FA, a phenolic acid, is a natural and safe antioxidant, approved by the Japan Food Chemical Research Foundation and the US Food and Drug Administration for use as a food additive in 2010 [3]. FA possesses several benefits, such as the delayed progression of fatty liver disease, improved vascular function [4], and anti-diabetic and anti-cancer activities [5]. To meet sustainable development objectives, biological catalysis for the purpose of degrading abundant biomass waste is promising [6]. For instance, FAE has been used with cellulase and hemicellulase for the production of fuel ethanol, fermentable sugar, and other high-value by-products [7,8]. Moreover, FAE has also been used in paper processing and bleaching, and as an additive in animal feed to improve nutrient digestibility [3].

Owing to their utility, strains that secrete FAEs continue to be identified, and some FAEs from fungi have been studied extensively, including their classification [7], biochemical properties, three-dimensional structures, and catalytic mechanisms [9]. However, there is the demand to identify more novel FAEs because of lower enzyme activity and poor thermal stability [7,10,11,12]. Gopalan et al. [10] pointed out that most FAEs were usually less than 1 U/mL of culture medium, while Meng et al. [12] mentioned that most FAEs were mesophilic enzymes, suggesting that they lose their catalytic activity when the temperature exceeds 50 °C. Natural environments and metagenomic libraries have been used to screen and identify novel FAEs [6,13]. To boost the industrial application and simplify the purification of FAEs to facilitate the efficient production of FA, genes are cloned in different hosts in order to overexpress recombinant enzymes [14]. *Escherichia coli*, a common and highly efficient expression system, is often a poor choice for industrial food production since it produces endotoxins and is not a “Generally Regarded as Safe” (GRAS) host. In recent years, an increasing number of researchers have begun to use *Bacillus subtilis* as a host to express enzymes, mainly because it is a food-grade microorganism and suitable for the industrial production of FAEs. *B. subtilis* is also amenable to a simple fermentation process and lacks obvious codon bias [15].

To date, there have been few studies on the heterogeneous expression of FAE using the *B. subtilis* system. In this study, the FAE from *B. pumilus* SK52.001 (BpFAE) was identified, cloned, and heterogeneously expressed in *B. subtilis* for the first time. The studies were conducted to characterize the biochemical properties and liberate FA from de-starched wheat bran (DSWB) by synergy action of BpFAE and commercial xylanase.

## 2. Materials and Methods

### 2.1. Chemicals and Reagents

Lysozyme, ampicillin, and kanamycin sulfate were purchased from Sangon Biotech Co., Ltd. (Shanghai, China). FA (content > 99%) was obtained from J&K Scientific Ltd. (Shanghai, China). Methyl ferulate (MFA) (content > 99%) was purchased from ThermoFisher Scientific (Shanghai, China). Commercial xylanase (10,000 U/g) was obtained from Macklin Co., Ltd. (Shanghai, China). Wheat bran was obtained from Auchan Supermarket (Wuxi, China). Other chemicals were of analytical grade and were purchased from Sinopharm Chemical Reagent Co., Ltd. (Shanghai, China).

### 2.2. Strain Screening

The soil samples from Wuxi, Jiangsu Province were suspended in sterile saline at a final concentration of 1 g/mL at 30 °C and 200 r/min for 30 min. The strains were isolated using gradient dilution in a non-selective isolation medium (200 g/L potato extract, 20 g/L source, and 20 g/L ager). Subsequently, the colonies were picked up from the selective medium (2 g/L NaNO_3_, 1 g/L K_2_HPO_4_, 0.5 g/L KCl, 0.5 g/L MgSO_4_·7H_2_O, 0.01 g/L FeSO_4_·7H_2_O, and 20 g/L ager), with ethyl ferulate (10 g/L) as the sole carbon source at 30 °C for 48 h. The resultant colonies surrounded by halo were purified in a non-selective isolation medium 3 times and inoculated in a fermentation medium (2 g/L NaNO_3_, 1 g/L K_2_HPO_4_, 0.5 g/L KCl, 0.5 g/L MgSO_4_·7H_2_O, 0.01 g/L FeSO_4_·7H_2_O, and 20 g/L wheat bran) at 30 °C, with shaking at 200 r/min for 26 h. The enzyme activity of FAE was estimated and the strain with the highest activity was identified as the target strain for further study.

### 2.3. Engineered Strain, Plasmid, and Culture Media

*E. coli* DH5α (Sangon Biotech, Shanghai, China) and *B. subtilis* WB800 (preserved in our laboratory) were hosts for cloning and expression, respectively. The shuttle plasmid pMA5 with *Nde*I and *Bam*HI sites (Talen Biotech, Shanghai, China) was used as the expression vector. Luria-Bertani (LB) broth (5 g/L yeast extract, 10 g/L tryptone, and 10 g/L NaCl) was prepared for the routine growth of *E. coli*. Ampicillin was dissolved in sterile water and used at a final concentration of 100 μg/mL. A super-rich medium (SR) containing 50 μg/mL kanamycin consisted of 3 g/L K_2_HPO_4_, 20 g/L yeast extract, 25 g/L tryptone, and 30 g/L glucose for *B. subtilis* WB800 cultivation.

### 2.4. Cloning

Genomic DNA from the *B. pumilus* SK52.001 strain was extracted using the Ezup Column Bacteria Genomic DNA Purification Kit (Sangon Biotech, Shanghai, China). The *fae* gene fragment was amplified with primers, Bp*fae* forward: AAAAGGAGCGATTTACATATGATGAACTTACAAGAGCAAATCAAAATCGCTGC and Bp*fae* reverse: GAGCTCGACTCTAGAGGATCCTTAATGGTGATGGTGATGATGTTCAAATGCCTTT. The FAE sequence fused with a 6 × histidine tag at C-terminus was ligated with the expression vector pMA5 between the *Nde*I and *Bam*HI sites, resulting in pMA5-*fae*. The recombinant plasmids were transformed into competent *E. coli* DH5α cells via heat shock. Positive transformants were cultured overnight in 5 mL LB medium supplemented with ampicillin, and plasmids pMA5-*fae* were extracted by FastPure Plasmid Mini Kit (Vazyme Biotech Co., Ltd., Nanjing, China) and sequenced by Sangon Biotech Co., Ltd. (Shanghai, China).

### 2.5. Heterogeneous Expression and Purification

For the expression of *fae*, the resultant plasmids pMA5-*fae* were introduced into *B. subtilis* WB800 to obtain the recombinant strains, *B. subtilis* WB800/pMA5-*fae*. A colony positive for kanamycin resistance was incubated in LB broth supplemented with 50 μg/mL kanamycin at 37 °C, with shaking at 200 r/min for 12 h as seed. To express the target enzyme, the engineered *B. subtilis* cells were cultured with 3% inoculation in 50 mL SR medium, with 50 μg/mL kanamycin at 37 °C, with shaking at 200 r/min for 14 h.

Following overexpression, all purification steps of the target protein were carried out at 4 °C. Cells expressing BpFAE were harvested by centrifugation at 8000× *g* for 15 min. The cells were resuspended in a sample buffer (50 mmol/L Tris-HCl, pH 8.0) containing 100 mmol/L NaCl and disintegrated by biological lysis with 20 mg/mL lysozyme in a water bath at 37 °C for 30 min, with gentle shaking every 10 min. Then, cells were ruptured by ultrasonication for 10 min (ultrasound 1 s, pause 2 s) and centrifuged at 6000× *g* for 15 min. To purify the enzyme, the nickel affinity column was washed with deionized water and pre-equilibrated with a binding buffer (500 mmol/L NaCl, 50 mmol/L Tris-HCl, pH 8.0). The supernatant was loaded into the column, which was then washed with a binding buffer and a washing buffer (500 mmol/L NaCl, 50 mmol/L Tris-HCl, 20 mmol/L imidazole, pH 8.0) to remove unbound or weakly bound proteins. Subsequently, the target enzyme was eluted with an elution buffer (500 mmol/L NaCl, 500 mmol/L imidazole, 50 mmol/L Tris-HCl, pH 8.0) and dialyzed against a sample buffer, with three buffer exchanges to remove imidazole.

### 2.6. Molecular Mass Determination

The subunit and native molecular mass (Mm) of BpFAE were evaluated by sodium dodecyl sulfate (SDS) and native polyacrylamide gel electrophoresis (PAGE, 12% separating gel), respectively. The protein was stained with Coomassie brilliant blue R250 and de-stained with a decoloring solution (ethanol:acetic acid:distilled water = 1:2:17).

### 2.7. Hydrolytic Activity Assay and Protein Concentration Determination

The hydrolytic activity of BpFAE was measured using MFA as substrate. The enzymatic reaction was conducted in a 1 mL mixture containing 990 μL MFA (3 mmol/L, dissolved in 50 mmol/L Tris-HCl, pH 9.0) and 10 μL enzyme solution at 50 °C for 10 min, and terminated by placing this mixture in a boiling water bath for 10 min.

The concentration of FA was analyzed with a high-performance liquid chromatography (HPLC) system (Agilent 1200, Agilent Technologies Inc., Santa Clara, CA, USA), equipped with an ultraviolet detector at 320 nm and a ZORBAX Eclipse Plus C18 column (Agilent, 150 mm × 4.6 mm, 3.5 μm). The injection volume was 10 μL, and the flow rate and temperature were controlled at 1 mL/min and 30 °C. The mobile phase consisted of 1% (*v/v*) acetic acid in H_2_O_dd_ (eluent A) and methanol (eluent B). The elution gradient protocol was: 0 min (90% A/10% B); 0–0.23 min (70% A/30% B); 0.23–1.66 min (50% A/50% B); 1.66–4.97 min (100% B); 4.97–5.57 min (85% A/15% B); 5.57–7.52 min (90% A/10% B). A standard curve was generated by linear fitting based on the retention time and FA peak area. One unit of FAE hydrolytic activity was defined as the amount of enzyme required to release 1 μmol FA per minute under standard assay conditions.

The protein concentration of a purified enzyme was evaluated using the Lowry method with bovine serum albumin as a standard [16].

### 2.8. Biochemical Characterization of BpFAE

To determine the optimal pH of BpFAE, experiments were carried out under five buffer conditions: acetate buffer (50 mmol/L, pH 4.0–5.5), 2–(N–morpholino) ethanesulfonic acid (MES) buffer (50 mmol/L, pH 5.5–6.5), sodium phosphate buffer (50 mmol/L, pH 6.5–7.5), Tris-HCl (50 mmol/L, pH 7.5–9.0), and glycine-NaOH buffer (50 mmol/Lol/L, pH 9.0–10.0). The relative activity was normalized to the maximum activity set at 100%. In order to study pH stability, the enzyme was pretreated at pH 8.5, 9.0, and 9.5, at 4 °C for 28 h. The activity at the beginning was set at 100%.

To investigate the optimal temperature, enzyme activity was assayed at different temperatures ranging from 35 to 90 °C. The highest enzyme activity was set at 100%. For thermal stability, the enzyme was incubated at 50, 55, 60, and 65 °C prior to the residual enzyme activity assayed. The activity of enzyme stored at 4 °C was specified as 100%. The half-life time and melting temperature (*T*_m_) were determined. The slope defined as *k* was obtained according to a linear fitting of the natural logarithm of the relative activity versus time. The half-life was calculated by ln2/*k*. The molar ellipticity at 222 nm of the enzyme was recorded as a function of variable temperature ranging from 25 to 90 °C on a circular dichroism spectropolarimeter (CD) (Chirascan V100, Applied Photophysics Ltd., Leatherhead, UK), with a bandwidth of 1.0 nm, sensitivity of 20 mdeg, heating rate of 0.2 °C/min, and a 0.1 cm quartz cuvette. The *T*_m_ was calculated by the acquisition of maximum slope.

### 2.9. Kinetic Parameters

The kinetic parameters of the purified BpFAE were measured at different concentrations of MFA (0.1–8 mmol/L) at 50 °C and pH 9.0. The *K*_m_, *V*_max_, and *K*_cat_ values were calculated by fitting the nonlinear Hill function using OriginPro 9.1 (Origin Lab Inc., Northampton, MA, USA).

### 2.10. Production of FA from DSWB

DSWB was incubated with 3 g/L potassium acetate at 95 °C for 1 h with constant stirring, followed by the removal of soluble starch using distilled water. Subsequently, the treated DSWB was dried to a constant weight at 55 °C for later use.

The total amount of FA in the DSWB above was chemically extracted by incubation in 2 mol/L NaOH at 50 °C, with shaking at 150 r/min for 4 h, and supplementing 2 mol/L HCl at 85 °C for 1 h. Then, the suspension was centrifuged at 8000× *g* for 10 min. The supernatant was analyzed by HPLC-UV, as described above.

The FA release from DSWB was carried out by enzymatic degradation. The 75 mg DSWB was suspended in 3 mL of the Tris-HCl buffer (50 mmol/L, pH 9.0), which was enzymatically degraded by adding 0.18 mg of purified BpFAE, 0.31 mg of commercial xylanase, and 0.18 mg of BpFAE along with 0.31 mg xylanase. It was conducted at 50 °C for 4 h, with shaking at 150 r/min. The reaction mixture was terminated by boiling for 10 min and centrifuged at 8000× *g* for 10 min. The analysis of the released FA amount was conducted by HPLC.

### 2.11. Statistical Analysis

The data are presented as the mean ± standard deviation (SD, *n* = 3). Statistical analyses were performed using OriginPro 9.1 (Origin Lab Inc., Northampton, MA, USA). 

## 3. Results and Discussion

### 3.1. Strain Screening and Identification

A total of 105 strains were obtained after isolation. Among them, eight strains possessed FAE activities, which are shown in Table 1. The strain with highest enzyme activity (0.195 U/mL) was identified as *Bacillus pumilus* SK52.001 by 16S rRNA sequence, which was deposited in the China Center for Type Culture Collection, with accession number M2020421.

### 3.2. Sequence Analysis

PCR sequencing results showed that the open reading frame of *B. pumilus* SK52.001 *fae* is composed of 912 bp. The nucleotide sequence was submitted to the GenBank database with the accession No. MW455110. BLASTn analysis indicated that the nucleotide sequence showed the highest similarity (97.42%) with carboxylesterase A from *B. pumilus* NCTC10337 (GenBank ID: LT906438.1). In addition, the nucleotide sequence was 96.64% identical with alpha/beta hydrolase from *B. pumilus* ZB201701 (GenBank ID: CP029464), 90.03% with esterase from *B. safensis* U14–5 (GenBank ID: CP015607), and 85.54% with alpha/beta hydrolase from *B. altitudinis* 11–1-1 (GenBank ID: CP054136).

The FAE protein from *B. pumilus* SK52.001 is composed of 303 amino acid residues without a signal peptide and has a theoretical isoelectric point (pI) and subunit Mm of 5.22 and 33,633.13 Da, respectively, on the ExPASy website (https://web.expasy.org/protparam/, accessed on 22 October 2020). The grand average of the hydropathicity of BpFAE was −0.286, indicative of a hydrophilic protein. Additionally, BpFAE contains a sole alpha/beta hydrolase fold with a fairly wide range, from residues 67 to 272 as a core domain, which was forecast on the Conserved Domain Database website (https://www.ncbi.nlm.nih.gov/cdd, accessed on 23 October 2020).

**Figure 1:**
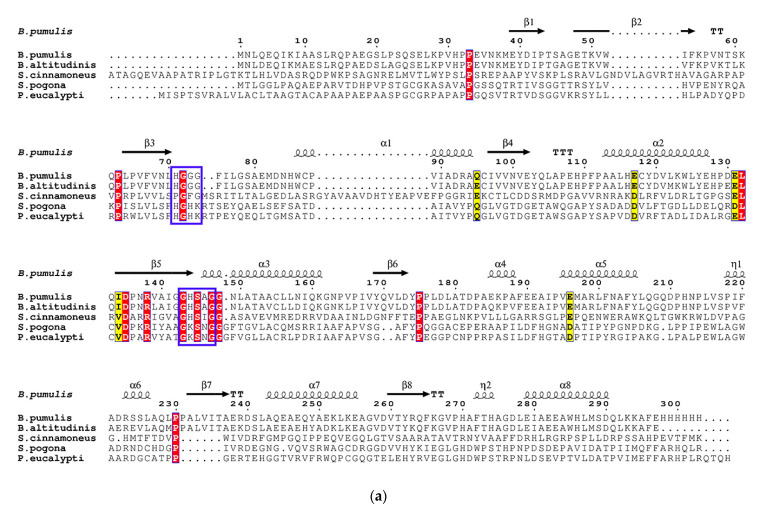

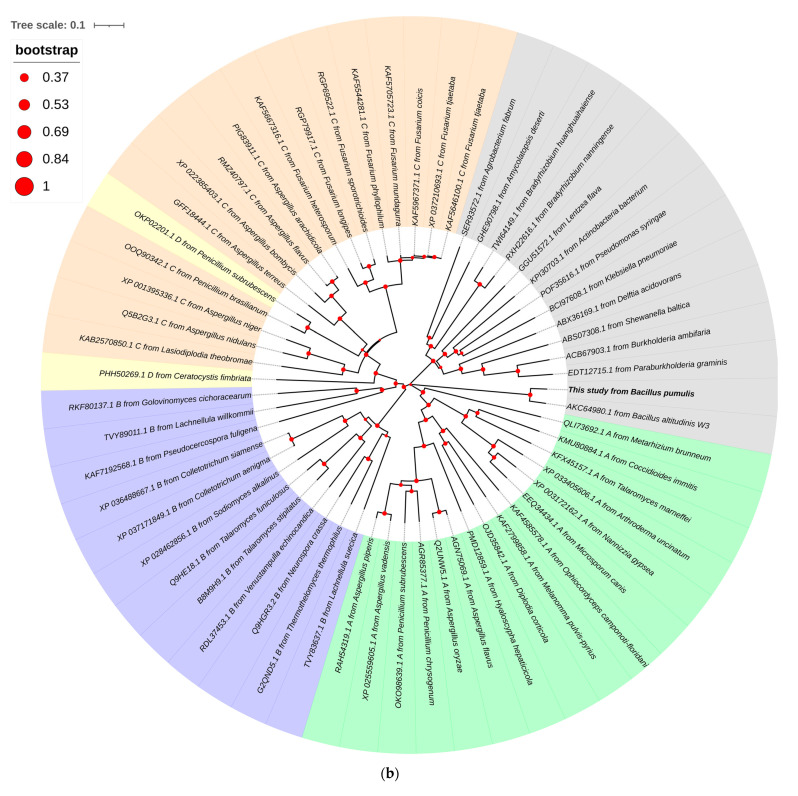
Sequence analysis of BpFAE. (**a**) The amino acid sequence of BpFAE from *B. pumilus* was aligned with BaFAE from *B. altitudinis* (GenBank ID: AKC64980), R18 from *Streptomyces cinnamoneus* (PDB: 5YAE), SpFAE from *S. pogona* (GenBank ID: WP_190824464), and PeFAE from *P. eucalypti* (GenBank ID: WP_185062649). The strictly conserved amino acids are displayed with a red background. The sequences with high similarity are shown against a yellow background. Two typical domains (GXSXG and HGGG) are indicated by a blue frame. (**b**) Phylogenetic tree based on a neighbor-joining algorithm using MEGA 7. Four types (A, B, C, D) and other FAEs from bacteria are highlighted in green, purple, orange, yellow, and grey, respectively.

The amino acid sequence homology analysis (Figure 1a) indicated that BpFAE exhibited the highest identity (89.90%) with the FAE protein from *B. altitudinis* (GenBank ID: AKC64980). However, BpFAE showed low identity (less than 50%) with FAEs from *Pseudonocardia eucalypti* (46.43%, GenBank ID: WP_185062649), *Saccharopolyspora pogona* (38.24%, GenBank ID: WP_190824464.1), *Paraphaeosphaeria minitans* (36%, GenBank ID: KAF9734932), and *Amycolatopsis umgeniensis* (23.56%, GenBank ID: WP_184905649). Nevertheless, BpFAE shared irrelative homology with FAEs from other *Bacillus* species, including *B. amyloliquefaciens* (GenBank ID: ANW61880, ANW61882, ANW61885), *B. megaterium* (GenBank ID: AIW07009, SFH00524), *B. aryabhattai* (GenBank ID: SDE01879), *B. timonensis* (GenBank ID: WP_010677557, WP_010677557), and *B. onubensis* (GenBank ID: WP_099362906). BpFAE contains a conserved motif GHSAG in line with the typical GXSXG motif of the alpha/beta hydrolases of the serine proteinase family.

The evolutionary relationship between BpFAE and other reported FAEs was evaluated on a phylogenetic tree based on the neighbor-joining algorithm using 1000 bootstrap replicates in MEGA 7 (Figure 1b). From Figure 1b, BpFAE was rooted into type A, which was distinguished from other bacterial FAEs. The known FAEs were classified into five types based on amino acid sequence identity and tested substrate specificity, A, B, C, D, and others [17]. Among the first four types, the typical GXSXG motif is conversed GHSLG with type A, GXSSG with type B, GCSTG with type C, and GWSYG with type D [18]. Combined with the phylogenetic tree (Figure 1b), however, BpFAE was subordinated to type A and different from the GHSLG motif.

### 3.3. Expression, Purification, and Molecular Mass Determination of Recombinant FAE

To date, several FAE genes have been successfully expressed and characterized in engineered strains, including *E. coli* [19], *Kluyveromyces marxianus* [20,21], *Pichia pastoris* [22,23], and *Trichoderma ressei* [1]. However, there is no report on the *B. subtilis* expression system of FAE. To explore the feasibility of *B. subtilis* as an expression system, *B. pumilus* SK52.001-derived *fae* was heterogeneously expressed in *B. subtilis* WB800, a safe and effective food-grade host. Because BpFAE was fused with 6× His-tags at the C-termini before construction, it was purified with a nickel-affinity column. After nickel-affinity column purification, the specific activity of the purified protein was 377.9 ± 10.3 U/(mg protein), which is the highest reported value (Table 2).

**Figure 2:**
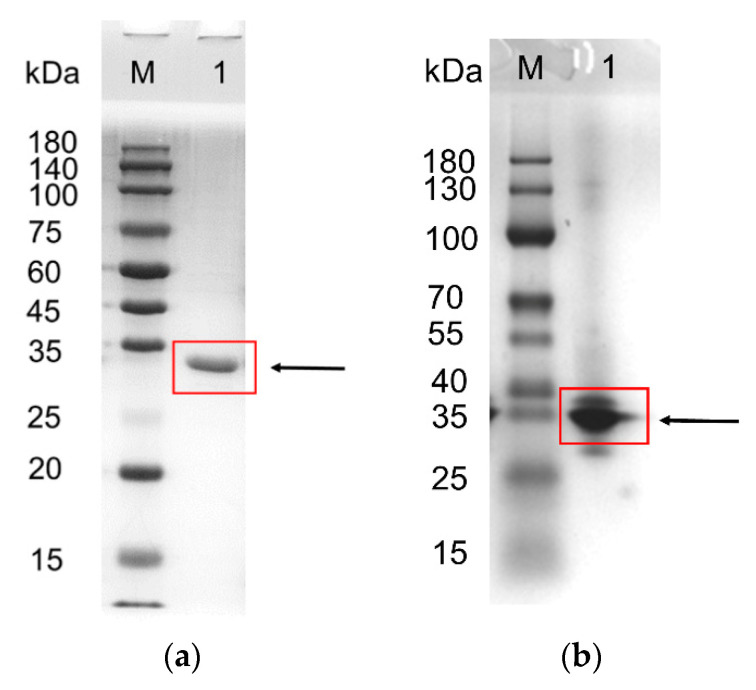
Analysis of molecular mass. (**a**) Sodium dodecyl sulfate polyacrylamide gel electrophoresis. (**b**) Native polyacrylamide gel electrophoresis. Lane M, the molecular mass of marker; lane 1, purified BpFAE.

As shown in Figure 2a, the subunit Mm was 32.6 kDa as evaluated by SDS-PAGE. The native Mm determined via native-PAGE was 33.6 kDa (Figure 2b), in line with the theoretical Mm (33,633.13 Da). These results indicate that BpFAE is a monomer, similar to FaeLcr from *Lactobacillus crispatus* [6]; however, it is different from BiFae1A and BiFae1B from *Bacteroides intestinalis,* which are tetramer and dimer as shown by structural analysis [26].

### 3.4. Effect of pH on Enzyme Activity and Stability

**Figure 3:**
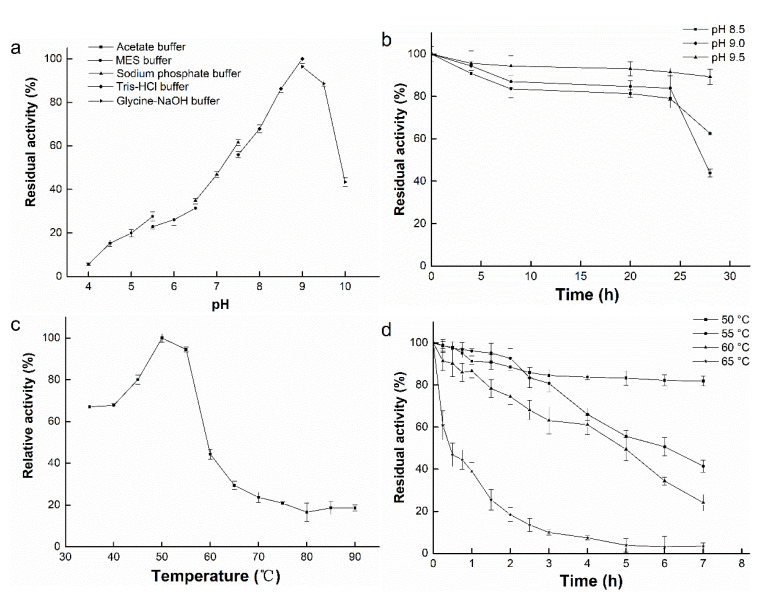
Properties of BpFAE. (**a**) Effect of pH. (**b**) Stability of pH. (**c**) Effect of temperature. (**d**) Thermal stability. The data were acquired in triplicate experiments.

As shown in Figure 3a, BpFAE exhibited the highest activity at pH 9.0, and 85% of the activity was retained at the pH of 8.5 or 9.5. However, the activity declined sharply under acidic conditions, suggesting that BpFAE is an alkaline enzyme and sensitive to acidic conditions. Moreover, these data indicate that BpFAE, with an optimal pH of 9.0, prominently differs from most FAEs (shown in Table 2) that have an optimal pH ranging from 5.0 to 8.0 [1,22,27,28]. In terms of pH stability, more than 80% of the enzyme activity was retained after incubation at pH 8.5, 9.0, and 9.5 for 28 h at 4 °C, respectively, as shown in Figure 3b. This result indicates that BpFAE is a relatively stable protein under alkaline conditions.

### 3.5. Effect of Temperature on Enzyme Activity and Stability

The effects of temperature on BpFAE were evaluated at pH 9.0 over a range of temperatures, from 35 to 90 °C. As shown in Figure 3c, the activity was the highest at 50 °C and the enzyme activity decreased sharply at temperatures exceeding 60 °C. The optimal temperature largely depended on the microbial sources. For most FAEs, such as those from the soil metagenomic library [13], *Lactobacillus fermentum* [19], *Bacteroides intestinalis* [26], *Aspergillus oryzae* [27], and *Penicillium purpurogenum* [29], the optimal temperature is typically low, ranging from 37 to 50 °C (Table 2). The same for FAEs from the thermophilic compost metagenomic library [30], which displayed a maximum activity at 41 °C, indicating that FAEs are sensitive to temperature.

**Figure 4:**
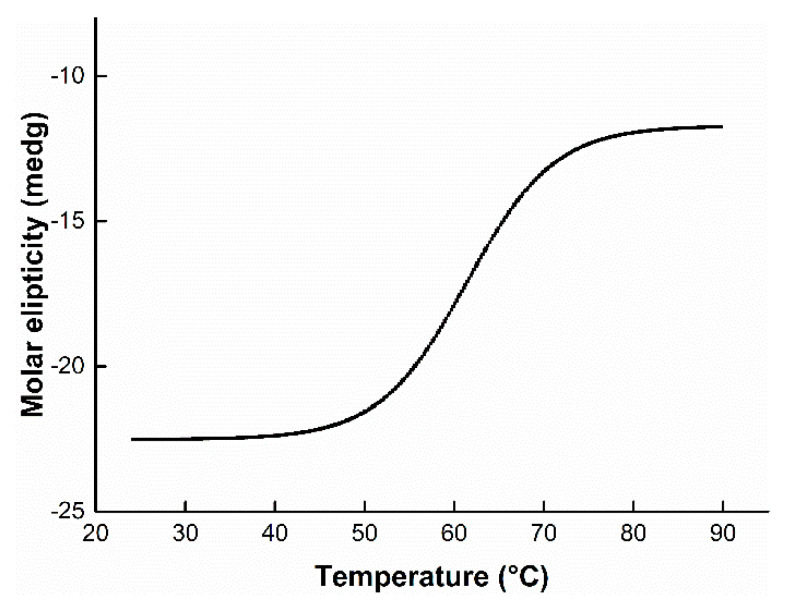
Thermal deformation curve at temperatures ranging from 25 to 90 °C.

For the assessment of thermal stability, BpFAE was pretreated at 50, 55, 60, and 65 °C. As shown in Figure 3d, about 80% residual activity was retained when the enzyme was incubated at 50 °C for 7 h. More than 50% activity was maintained at 55 and 60 °C for 5 h. However, there was only 18% residual enzyme activity after pretreating for 2 h at 65 °C, and hydrolytic activity was completely lost after 5 h at 65 °C. Previous studies on FAEs have exhibited similar thermal stability. For instance, FAE from *Lactobacillus crispatus* retained 60% and 10% residual activity at 60 and 65 °C, respectively, after incubation for 1 h [6]. A vast majority of the enzymes display drastic decreases in activity after pre-incubation at 60 °C for 0.5 h [1]. However, BpFAE could retain 60% residual activity after incubation at 60 °C for 4 h, suggesting that BpFAE showed good thermal stability. The half-lives of BpFAE at 50, 55, 60, and 65 °C were 1434, 327, 235, and 68 min, respectively. Notably, FAE from *Penicillium piceum* has been shown to exhibit the highest thermal stability of 220 min at 70 °C and 150 min at 60 °C [18]. In general, BpFAE exhibited good thermal stability. The *T*_m_ of BpFAE was 61.3 °C estimated at 222 nm using CD with single-point temperature changes (Figure 4).

### 3.6. Kinetic Parameters

The kinetic parameters were obtained by fitting the Hill function under various substrate concentrations (0.1–8 mmol/L). The *K*_m_, *V*_max_, and *K*_cat_ values were 0.95 mmol/L, 0.22 μmol/(L·min), and 514.91 1/s, respectively. BpFAE showed a lower *K*_m_ value for MFA as compared to FAEs from *Eupenicillium parvum* (6.24 mmol/L) [24], indicating a stronger affinity between BpFAE and its substrate. The *K*_m_ of BpFAE was similar to those of BaFae04 and AnFaeA from *B. amyloliquefaciens* and *A. niger* (1.14 mmol/L and 1.4 mmol/L, respectively) [11,28]. The catalytic efficiency (*K*_cat_/*K*_m_) of MFA was 542.01 mmol/(L·s), reflecting a high substrate conversion rate. Compared to the catalytic efficiency of FAE from *B. intestinalis* (321.9 mmol/(L·s) [26] from the soil metagenomic library (79.04 mmol/(L·s)) [13], and *A. terreus* (254.9 mmol/(L·min)) [25], BpFAE was more efficient (1.68×, 6.86×, and 127.58×, respectively).

### 3.7. Release FA from DSWB

**Figure 5:**
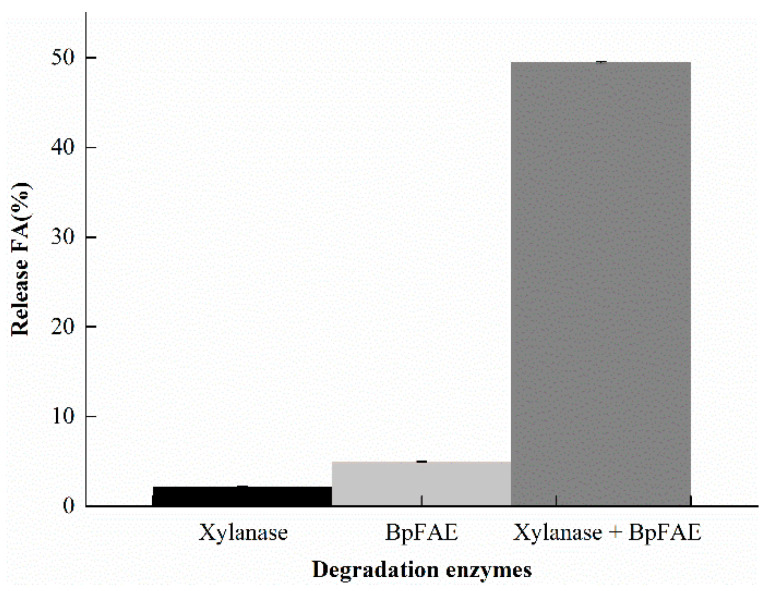
Production of FA from DSWB using different enzymes.

The enzymes, especially FAE, were used to produce FA from biomass, since FA was linked to lignin or arabinoxylans in the plant cell walls [31,32,33]. Several studies indicated that approximately 70% FA in DSWB was easily disrupted as free FA after enzymatic catalysis [11,24]. After the alkali-acidic breakage of DSWB, the total FA amount was calculated as 3.91 mg/g DSWB. Long et al. [24] showed the similar result that the alkali-extractable FA from DSWB was 4.04 mg/g, indicating that the extractable method in this study is feasible and effective. When the purified BpFAE alone was used to catalyze the breakage of chemical bonds and release FA, it was observed that only 5% of the soluble FA was liberated from DSWB at 50 °C for 4 h. It is mainly because the xylan backbone with a large molecular weight becomes a hindrance, resulting in the lower catalytic efficiency of BpFAE. Xu et al. [6] could liberate a maximum FA amount of 199 μg from 0.2 g DSWB using 2.0 mg of purified Faelcr alone at 55 °C for 8 h. It suggests that the hydrolysis of DSWB using BpFAE is much more efficient. Meanwhile, only 2% of FA was released from DSWB using commercial xylanase. It is probable that the structure of DSWB was partially damaged in the alkaline buffer system. Several studies have shown that the synergistic action of FAE and xylanase can significantly enhance the quantity of FA [34,35,36]. In this study, the same result was revealed, as shown in Figure 5. The amount of FA was reached at 49.47% in the presence of BpFAE and xylanase, increasing about 10 times using BpFAE alone. Xylanase cleaves the breakage of glycosidic bonds between xyloses in the xylan backbone, leading to short-chain ferulated oligosaccharides [37]. It is more susceptible to the combination with BpFAE, which boosts production of FA. These results reflect that BpFAE prefers lower molecular-weight natural substrates [13]. Wu et al. [11] liberated FA from DSWB by synergistic action of AnXyn11A and AnFaeA, reaching 70% and increasing about 4.2 times as compared to AnFaeA alone (from 16.8% to 70%). In accordance with this study, Mkabayi et al. [34] also employed termite metagenome-derived FAE along with Xyn11 from *Thermomyces lanuginosus* to greatly increase the amount of FA from corn cobs. Combined with the previous results, FAE along with xylanase is a reasonably applicable approach to produce FA from biomass.

## 4. Conclusions

In this study, an alkaline FAE from *Bacillus pumilus* SK52.001 was identified and heterogeneously expressed in *B. subtilis* WB800 for the first time. The enzyme exhibited an optimal alkaline pH and good thermal stability. Additionally, high specific activity was observed in this study, which was up to 377.9 ± 10.3 U/mg of protein using MFA as a substrate. The production of FA from DSWB was greatly improved to 49.47% using purified BpFAE in the presence of commercial xylanase, compared to 5% of FA production using BpFAE alone. In summary, BpFAE adopting the *B. subtilis* expression system is an efficient and promising biocatalyst. It proves that FAEs based on the recombinant *B. subtilis* may be conceivable for waste biomass degradation to promote the production of FA from food by-products, as a response to sustainable development.

## Figures and Tables

**Table 1:** FAE activities of strains.

Number	FAE Activity (U/mL)	Number	FAE Activity (U/mL)
2	0.034	55	0.019
3	0.195	68	0.022
9	0.161	72	0.170
48	0.050	73	0.160

**Table 2:** Comparison of properties of different FAEs.

Organisms	Subunit (kDa)	Optimal pH	Optimal Temperature (°C)	Thermal Stability	Km (mmol/L)	Reference
*Aspergillus niger*	56	5	50	NR	NR	[1]
*Lactobacillus crispatus*	28	7	65	60% (60 °C 1 h)	NR	[5]
soil metagenomic library	38.8	8	37	10% (55 °C, 1 h)	0.467	[8]
*Penicillium piceum*	56	3	70	50% (70 °C, 220 min)	NR	[16]
*Bcteroides intestinalis*	71.1	7.5	37	NR	0.35	[20]
*Bacillus amyloliquefaciens*	NR	8	40	60% (60 °C, 1 h)	1.14	[24]
*Aspergillus niger*	40	5	45	50% (55 °C, 30 min)	1.4	[25]
*Bacillus pumilus*	33	9	50	50% (65 °C, 63 min)	0.95	This study

NR: Not reported.
